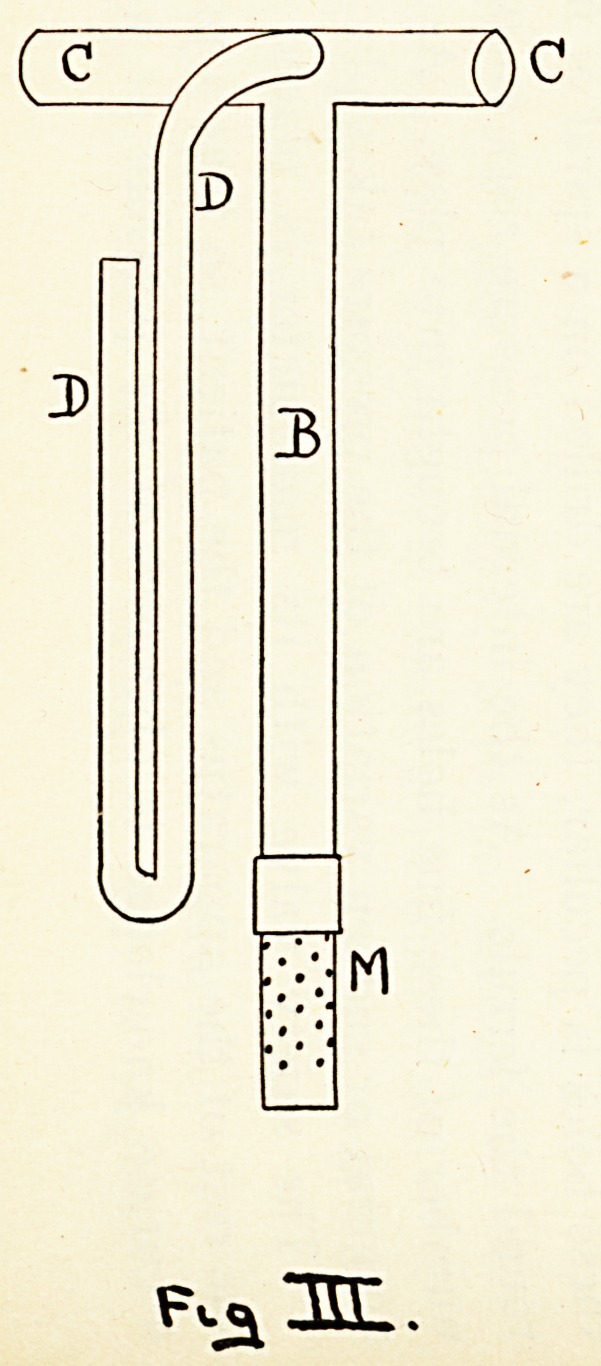# A Combined Manometer and Safety Valve for Intratracheal Anæsthesia

**Published:** 1913-12

**Authors:** Stuart V. Stock, J. D. Fry

**Affiliations:** Senior Assistant Anæsthetist, Bristol Royal Infirmary; Anæsthetist, Bristol Eye Hospital; Lecturer and Demonstrator in Physics, University of Bristol.


### A COMBINED MANOMETER AND SAFETY VALVE FOR INTRATRACHEAL ANÆSTHESIA.

BY

STUART V. STOCK, M.B., B.S., F.R.C.S.,
*Senior Assistant Anæsthetist, Bristol Royal Infirmary; Anæsthetist, Bristol Eye Hospital,*

AND

J. D. FRY, B.Sc.,
*Lecturer and Demonstrator in Physics, University of Bristol.*

---

THE method of Meltzer and Aner, adapted for use in the human subject by Elsberg, makes an advance in our methods of inhalation anæsthesia, the value of which in certain operations cannot be over estimated. In this method the anæsthetist is to a large extent dependent on his apparatus. Too much care, therefore, cannot be expended on its design and construction.

The various excellent pieces of apparatus on the market appeared to the writers to fall short in some respect or other of the ideal machine, and with a view to producing an instrument capable of accurate adjustment and dosage, at the same time simple in construction, and as far as possible reliable in its action, they started experimenting in March of this year.

The first piece of the apparatus to be taken in hand was the safety valve. Those familiar with the intratracheal method of administering anæsthetics well know that an efficient safety valve is a *sine qua non.* Two types of valve appear to be in use, In the first of these the pressure is regulated by a spring, while in the second the open end of a glass tube is immersed in mercury. the pressure being regulated by the depth to which the tube is immersed.

The first type was abandoned, both on account of the difficulty of obtaining a suitable accurate adjustment, and of its uncertainty of action due to occasional stiction of the working parts which renders it unreliable.

The second type was more promising. It is invariably certain in its action, and the " blow-off pressure " can be regulated to a nicety. Experiments were made with a view to constructing a manometer and safety valve combined, by means of which the pressure should be under observation and control, so that the excess pressure beyond a determined point should automatically blow off rapidly and steadily, without any great variation of the internal pressure.

The instrument in its final form consists of a bottle (*A*) holding the mercury. Its neck is fitted with a perforated cork (*N*), and from its side springs a vertical tube (*E*), through which the air escapes. Through the perforated cork (*N*) passes the vertical tube (*B*), to the lower end of which is attached a steel ferrule (to be afterwards described). At its upper end it communicates with the tube (*C*), through which the main stream of air and ether passes on its way to the lungs. Springing from the junction of (*B*) with (*C*) is the manometer tube (*D*) (see Fgs. I and 3). The tube (*B*) is a sliding fit in the cork (*N*) of the bottle (*A*). Behind the mercury bottle stands a pillar (*P*), which carries a guide (*H*), in which slides the rod (*G*), actuated by a rack and pinion movement (*K*). The upper end of (*G*) carries two arms (*FF*) (see Fgs. I and 2), whose ends embrace the tube (*C*). Thus by a movement of the wheel (*K*) the tube (*B*) can be raised or lowered, and the steel ferrule on its end immersed to a greater or less depth in the mercury. This arrangement allows a very fine adjustment of pressure with the very greatest ease.

A feature of the safety valve is the ferrule (*M*) (Fg. 3). It will be noted that the ferrule (*M*) made of steel, and cemented to the end of (*B*), is perforated by a large number of fine holes. The combined area of these small holes is slightly greater than the cross sectional area of the tube (*B*). The arrangement of these holes is peculiar, they are drilled on a spiral of fine pitch round the ferrule. As the internal pressure rises an increasing number of these fine holes are brought into play. As a result, no great or sudden variation of the pressure takes place.

The safety valve with its manometer is placed between the rest of the apparatus and the patient, so that a practically accurate knowledge is obtained of the pressure at which the

Fig III.

Fig II.

Fig. I.

mixture of air and ether enters the lungs. A slight fall in pressure takes place between the safety valve and the eye of the tracheal catheter, owing to the frictional resistance of the tubing through which it passes, but this is so small that for practical purposes it can be disregarded. When not in use the open ends of all the tubes are closed with corks, thus rendering it portable. The safety valve has been in use clinically since April last, and has given complete satisfaction. A description of it is published now before the completion of the machine, in the hope that it may prove of use to other workers in the field.

Quantitative experiments have been made on the cooling of the ether air mixture due to the evaporation of ether, for the purpose of devising a more convenient vaporiser. Other experiments are now in progress dealing with the construction of a simple apparatus for accurately determining the percentage of ether in a given air ether mixture.

## Figures and Tables

**Fig. I f1:**
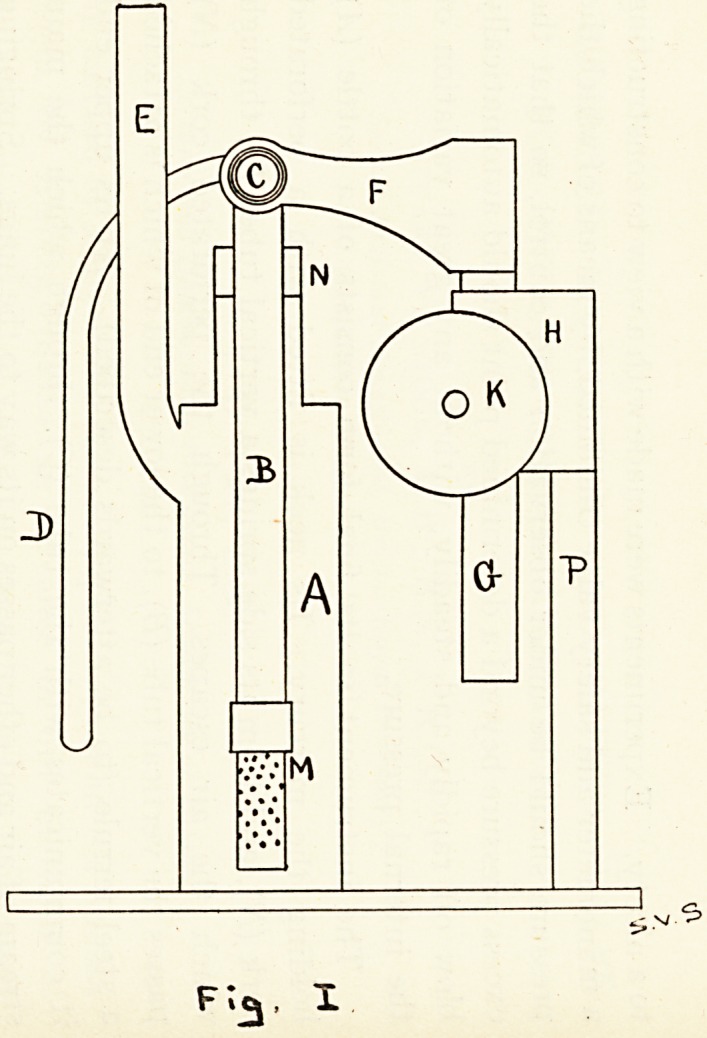


**Fig II. f2:**
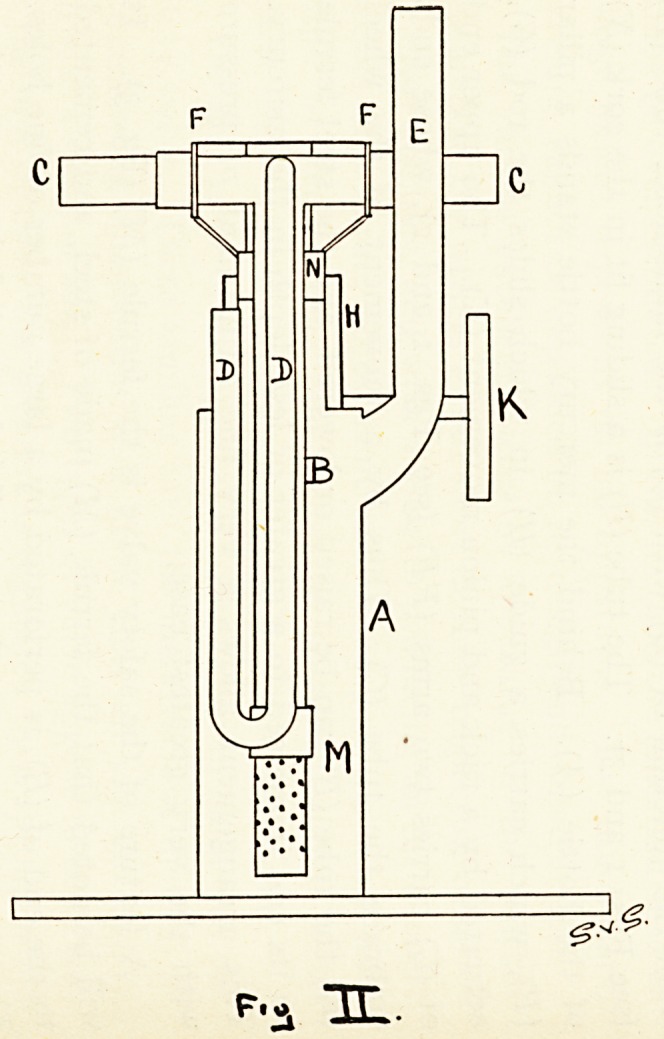


**Fig III. f3:**